# Comprehensive analysis of The Cancer Genome Atlas reveals a unique gene and non-coding RNA signature of fibrolamellar carcinoma

**DOI:** 10.1038/srep44653

**Published:** 2017-03-17

**Authors:** Timothy A. Dinh, Eva C. M. Vitucci, Eliane Wauthier, Rondell P. Graham, Wendy A. Pitman, Tsunekazu Oikawa, Mengjie Chen, Grace O. Silva, Kevin G. Greene, Michael S. Torbenson, Lola M. Reid, Praveen Sethupathy

**Affiliations:** 1 Curriculum in Genetics and Molecular Biology, Chapel Hill, NC, USA; 2 MD/PhD Program, Chapel Hill, NC, USA; 3 Department of Genetics, Chapel Hill, NC, USA; 4 Post-baccalaureate Research Education Program, Chapel Hill, NC, USA; 5 Department of Cell Biology and Physiology and Program in Molecular Biology and Biotechnology, Chapel Hill, NC, USA; 6 Lineberger Comprehensive Cancer Center, University of North Carolina at Chapel Hill, Chapel Hill, NC, USA; 7 Department of Laboratory Medicine and Pathology, Mayo Clinic, Rochester, MN, USA; 8 Department of Biostatistics, Chapel Hill, NC, USA; 9 Curriculum in Bioinformatics and Computational Biology, Chapel Hill, NC, USA; 10 Department of Pathology and Laboratory Medicine, University of North Carolina at Chapel Hill, Chapel Hill, NC, USA

## Abstract

Fibrolamellar carcinoma (FLC) is a unique liver cancer primarily affecting young adults and characterized by a fusion event between *DNAJB1* and *PRKACA*. By analyzing RNA-sequencing data from The Cancer Genome Atlas (TCGA) for >9,100 tumors across ~30 cancer types, we show that the *DNAJB1-PRKACA* fusion is specific to FLCs. We demonstrate that FLC tumors (n = 6) exhibit distinct messenger RNA (mRNA) and long intergenic non-coding RNA (lincRNA) profiles compared to hepatocellular carcinoma (n = 263) and cholangiocarcinoma (n = 36), the two most common liver cancers. We also identify a set of mRNAs (n = 16) and lincRNAs (n = 4), including *LINC00473*, that distinguish FLC from ~25 other liver and non-liver cancer types. We confirm this unique FLC signature by analysis of two independent FLC cohorts (n = 20 and 34). Lastly, we validate the overexpression of one specific gene in the FLC signature, carbonic anhydrase XII (CA12), at the protein level by western blot and immunohistochemistry. Both the mRNA and lincRNA signatures support a major role for protein kinase A (PKA) signaling in shaping the FLC gene expression landscape, and present novel candidate FLC oncogenes that merit further investigation.

Fibrolamellar carcinoma (FLC) is a rare form of liver cancer, representing ~1% of known liver cancer cases, and primarily occurring in adolescents and young adults without any history of liver disease, steatosis, fibrosis, or cirrhosis^1^,^2^,^3^,^4^. Histologically, FLC is unique among liver tumors, as it is characterized by intratumoral lamellar bands of fibrosis, large polygonal cells, eosinophilic cytoplasm, prominent nucleoli, and reduced nuclear to cytoplasmic ratio^1^,^2^,^3^. Currently, no clinical biomarkers for FLC exist and the majority of patients have metastases at the time of diagnosis. Surgery is the first-line therapeutic for FLC, but tumors often recur even following resection^5^,^6^. Patients with inoperable tumors have very poor prognosis and currently no standard accepted chemotherapeutic regimen for FLC exists^3^,^5^,^6^.

In addition to histology, several other features distinguish FLC from other liver tumors. The average age of onset for FLC patients is ~25 compared to approximately 60 for hepatocellular carcinoma (HCC) and cholangiocarcinoma (CCA) patients^1^,^2^,^3^,^5^,^7^,^8^. While HCC and CCA predominantly affect males, FLC has a relatively balanced gender distribution^1^. FLC patients typically do not exhibit dramatically elevated levels of aspartate aminotransferase (AST) or alanine aminotransferase (ALT), canonical markers of liver inflammation and necrosis^1^,^2^,^3^. In contrast, HCC commonly occurs in the context of long-standing cirrhosis often due to chronic hepatitis or alcoholic liver damage^8^. For this reason, FLC patients generally have more favorable survival compared to HCC and CCA patients. However, after controlling for the presence of cirrhosis, FLC and HCC patients have similar 5-year survival rates^9^. Finally, recurrent mutations commonly found in HCC (e.g., *TERT, TP53, CTNNB1*) or CCA (e.g., *KRAS, IDH1*) have not been identified in FLC tumors^2^,^3^,^10^,^11^.

FLC tumors express markers of biliary and hepatocytic, (e.g. CK7, HepPar-1) as well as neuroendocrine (e.g. PCSK1, NTS) lineages^3^,^11^,^12^,^13^,^14^,^15^. We also have shown that FLCs are remarkably rich in cancer stem cells, unlike HCCs and CCAs, and that their gene expression profile more closely resembles that of primitive biliary tree stem cells than any other lineage stage of the liver^16^. Recently, a heterozygous ~400 kb deletion on chromosome 19 was described in a study of 11 FLC patients^17^. This deletion was present in all FLC patient tumors, but not in adjacent non-tumor liver, and uniformly led to the chimeric transcript *DNAJB1-PRKACA*. This chimera swaps exon 1 of *PRKACA*, the catalytic subunit of protein kinase A (PKA), with exon 1 of *DNAJB1*, a heat shock protein of the HSP40 family. The *DNAJB1-PRKACA* transcript is translated into a fusion protein that retains PKA kinase activity^13^,^17^,^18^,^19^. Follow-up studies have detected this fusion in larger cohorts of FLC patients and have proposed that it may be a specific marker of FLC^11^,^20^.

Long intergenic non-coding RNA (lincRNA) is a class of non-coding RNAs longer than 200 nucleotides that do not code for protein and are key players in tumorigenesis^21^. Many lincRNAs are highly tissue and cell-type specific and have been shown to control proliferation and metastasis by their diverse cellular roles including transcriptional regulation, chromatin remodeling, intracellular protein localization, and competitive endogenous RNA activity^21^,^22^,^23^,^24^,^25^. Despite the growing appreciation for lincRNAs in cancer, they have not yet been examined in the context of FLC.

Recently, several groups have compared gene expression and mutation signatures of FLC tumors with that of adjacent non-tumor liver^10^,^11^,^13^,^15^,^17^. Other studies of specific genes have also revealed differences between FLC and HCC^26^,^27^,^28^,^29^,^30^. However, no study has yet characterized a molecular signature of FLC that distinguishes it from other liver cancers or other non-liver tumor types. Here, we identify six cases of FLC within The Cancer Genome Atlas (TCGA), half of which were originally classified as HCC. Transcriptomic analysis of FLCs reveals a unique messenger RNA (mRNA) and lincRNA signature of FLC. We confirm this signature in two independent validation cohorts of 20 and 34 FLC tumors. Most of the genes, and all of the lincRNAs, of the signature have not previously been specifically highlighted as markers of FLC, and therefore represent both novel markers and candidate oncogenes of FLC. A key finding is that many genes and lincRNAs in the FLC signature, including *LINC00473*, point to the likely importance of cyclic adenosine monophosphate (cAMP)-mediated PKA signaling in shaping FLC gene expression. We perform additional experimental validation for one member of the mRNA signature, carbonic anhydrase XII (*CA12*), which may have tumor and/or metastasis promoting functions. This work motivates future experimental investigation to determine the role of FLC signature genes and lincRNAs, as well as DNAJB1-PRKACA, in FLC pathogenesis.

## Results

### Identification of FLC cases in TCGA

**Figure 1:**
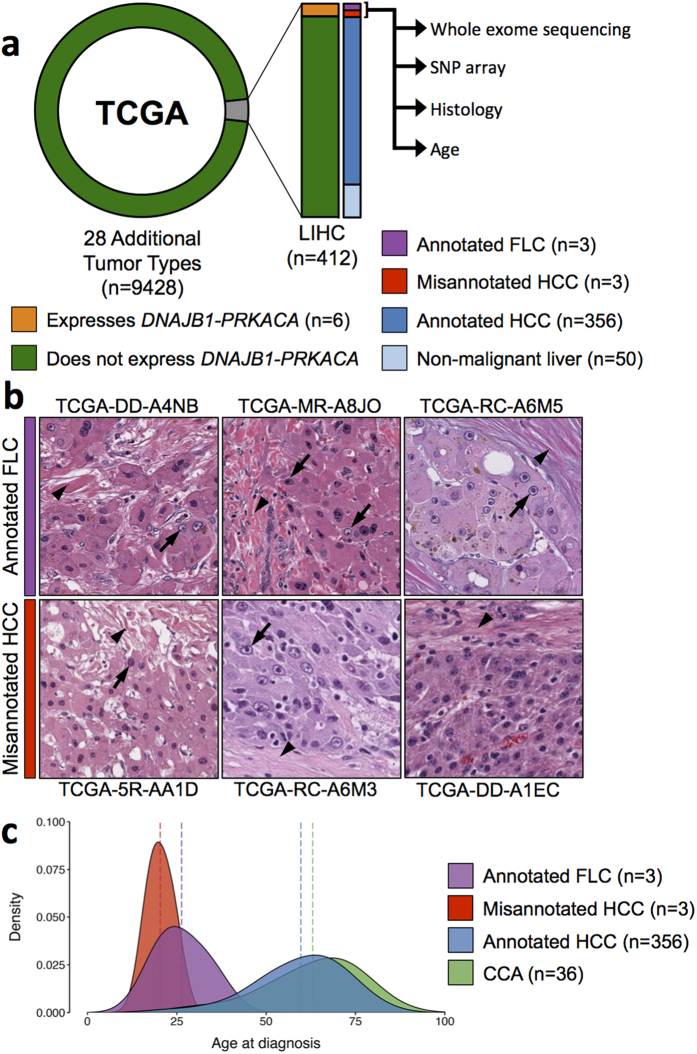
Identification of FLC samples in The Cancer Genome Atlas (TCGA). (**a**) Six FLC samples were identified in TCGA by RNA expression of *DNAJB1-PRKACA*. While all six samples were annotated as Liver Hepatocellular Carcinoma (LIHC), three were subannotated as FLC and 3 were subannotated as HCC. These three samples were examined further using whole exome sequencing, SNP array (to validate the chromosome 19 deletion resulting in *DNAJB1-PRKACA*), histology, and patient ages. (**b**) Sample histology images from the three annotated FLC samples and three misannotated HCC samples, all of which express *DNAJB1-PRKACA*. Arrowheads depict intratumoral fibrotic bands and arrows depict distinct nuclei with prominent nucleoli characteristic of FLC tumor cells. (**c**) Density plot depicting the age distribution of annotated FLC, misannotated HCC, annotated HCC, and CCA patients in TCGA. Dotted lines represent the mean age of each group.

The fusion transcript *DNAJB1-PRKACA* is thought to be present in almost all FLC cases^11^,^17^, but thus far has not been detected in any other type of liver cancer, leading to the hypothesis that *DNAJB1-PRKACA* is specific to FLC^20^. To evaluate this hypothesis, we analyzed RNA-sequencing (RNA-seq) data from 9158 tumor and 692 non-tumor samples across 29 different cancer types in TCGA (Supplementary Table S1). We detected robust evidence for expression of *DNAJB1-PRKACA* in six samples, all of which were tumors classified as Liver Hepatocellular Carcinoma (LIHC, Fig. 1a). Interestingly, three of these samples were subannotated as FLC, whereas the other three were subannotated as HCC. To validate the fusion in each of the samples, we confirmed the presence of the heterozygous ~400 kb deletion by analysis of whole exome sequencing (WES) data, single nucleotide polymorphism microarray (SNP array) data, or both (Supplementary Fig. S1). The three samples subannotated as HCC could be indicative of non-FLC tumors expressing *DNAJB1-PRKACA* (which has not been observed previously) or FLC tumors that were misannotated as HCCs (which was not uncommon historically). To distinguish between these two possibilities, we examined additional data sources available through TCGA for all six tumors expressing the chimera.

We first examined histology slides available for the six tumors expressing *DNAJB1-PRKACA*. Two liver pathologists (KGG, MST) independently reviewed all histology slides. The three samples annotated as FLC (TCGA-DD-A4NB, TCGA-RC-A6M5, TCGA-MR-A8JO) all showed classical features of this tumor type including lamellar bands of fibrosis, eosinophilic cytoplasm, large nuclei, and prominent nucleoli. Of the three samples annotated as HCC, one displayed classical histological features of FLC (Fig. 1b, TCGA-5R-AA1D), while the other two exhibited histological features of both FLC and HCC (Fig. 1b, TCGA-RC-A6M3, TCGA-DD-A1EC). The percentage of reads overlapping the *DNAJB1-PRKACA* fusion junction and the percentage of paired reads encompassing the entire junction were not significantly different in the samples with classical FLC histology compared to those with characteristics of both FLC and HCC morphology (Supplementary Fig. S1).

Due to the lack of access to the original tissues, we were not able to perform additional diagnostic staining of FLC protein markers (e.g., CD68, CK7) in order to further characterize the mixed FLC-HCC samples. However, we did analyze the expression of previously reported FLC RNA markers, *PCSK1*^13^,^14^,^15^ and *AGR2*^27^, and found that together they do differentiate the six *DNAJB1-PRKACA* expression tumors from all HCC and CCA samples (Supplementary Fig. S2).

We next examined the ages of the patients from which these six tumors were resected. The mean ages at diagnosis for annotated FLC and misannotated HCC patients were 26.33 (range of 20–34) and 20.33 (range of 17–24), respectively, both of which are consistent with the age of onset for FLC and significantly lower than that of HCC and CCA (Fig. 1c, Supplementary Fig. S1). Taken together with our histological and RNA marker analyses, we conclude that all six tumor samples expressing *DNAJB1-PRKACA* display features consistent with FLC. We refer to these six as FLC going forward.

### Identification of differentially expressed genes in FLC compared to HCC and CCA

**Figure 2:**
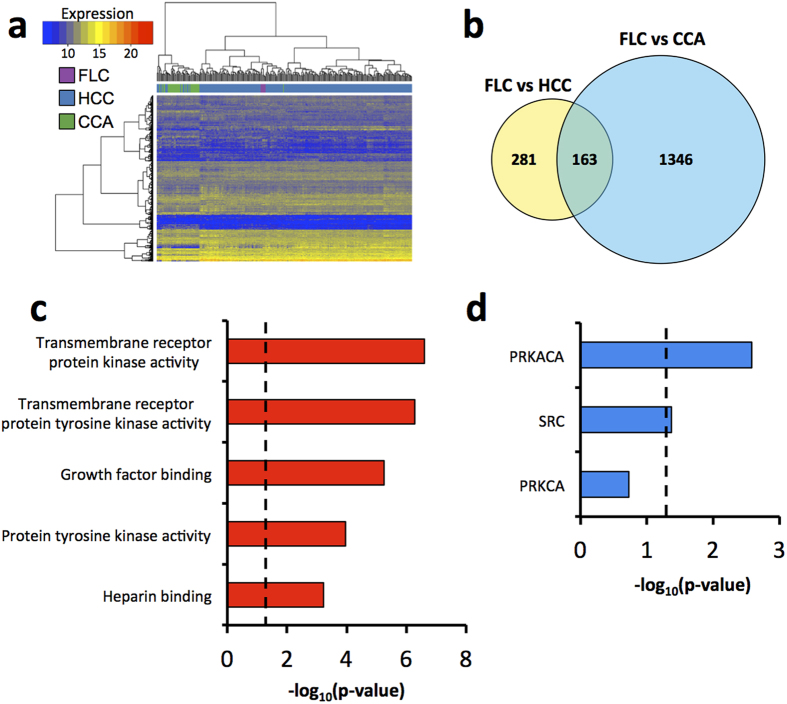
FLCs share a unique mRNA expression profile compared to other liver cancers. (**a**) Hierarchical clustering of FLC (n = 6), HCC (n = 263), and CCA (n = 36) samples in TCGA. Clustering was performed using the 10,000 most variable genes across all tumors following Variance Stabilizing Transformation with Euclidian distance and Ward’s minimum variance method. (**b**) Venn diagram showing differentially expressed genes between FLC and both HCC and CCA. Genes were defined as differentially expressed using the following criteria: average normalized counts ≥50 in at least one tumor type, fold change >2, and FDR < 0.05. (**c**) Gene Ontology Molecular Function analysis of the 163 genes differentially expressed between FLC and both HCC and CCA. (**d**) Kinase Enrichment Analysis of the 163 genes differentially expressed between FLC and both HCC and CCA. (**c**,**d**) Show only results with at least 5 genes in each category. Dotted lines represent p = 0.05. PRKACA (protein kinase A catalytic subunit alpha), PRKCA (protein kinase C alpha).

To determine if the gene expression profiles of FLCs differ from that of other liver cancers, we performed hierarchical clustering of FLC (n = 6), HCC (n = 263), and CCA (n = 36) samples with TCGA RNA-seq data using the 10,000 most variable genes across all samples. Our analysis revealed that the six FLCs clustered together in a clade separate from all HCCs and CCAs (Fig. 2a), indicating that FLCs have a unique gene expression profile distinct from other liver cancers. Importantly, all six FLC tumors were sequenced in separate batches. This finding held true with the addition of non-tumor liver (n = 50) and cholangiocyte (n = 9) samples to the analysis (Supplementary Fig. S3). It is important to note that FLC tumor purity^31^,^32^ was well within the range observed for HCC (Supplemental Fig. S4), indicating that tumor purity alone cannot account for the clustering of FLC samples.

Next we sought to identify the genes significantly differentially expressed between FLC and both HCC and CCA. Genes were considered in the analysis if they had an average normalized count ≥50 in at least one tumor type, and were defined as differentially expressed if they exhibited >2 fold-difference in average expression level and false discovery rate (FDR) < 0.05 between FLC and both HCC and CCA. We found 444 differentially expressed genes between FLC and HCC and 1509 between FLC and CCA. Of these, 163 were differentially expressed between FLC and both HCC and CCA with concordant directionality (Fig. 2b, Supplementary Table S2). As expected, hierarchical clustering of FLC, HCC, and CCA samples based on the expression of these 163 genes resulted in a dendrogram with a clade containing only the six FLC samples (Supplementary Fig. S5). Moreover, principal component analysis showed that these 163 genes distinguish FLC from each of 20 other non-liver cancer types for which gene expression data is available through TCGA (Supplementary Fig. S6), although there are some notable exceptions of specific papillary renal cell carcinoma tumor samples that cluster closely with the FLC cases (Supplementary Fig. S6i).

Gene ontology analysis of these 163 genes revealed a significant enrichment in transmembrane receptor protein kinase activity and growth factor binding (Fig. 2c). Furthermore, kinase enrichment analysis revealed that the 163 genes are most significantly over-represented for substrates of PRKACA, supporting the notion that wild-type PKA and/or DNAJB1-PRKACA may be critical to the etiology and molecular characteristics of FLC (Fig. 2d).

### Determination of an FLC mRNA signature

**Figure 3:**
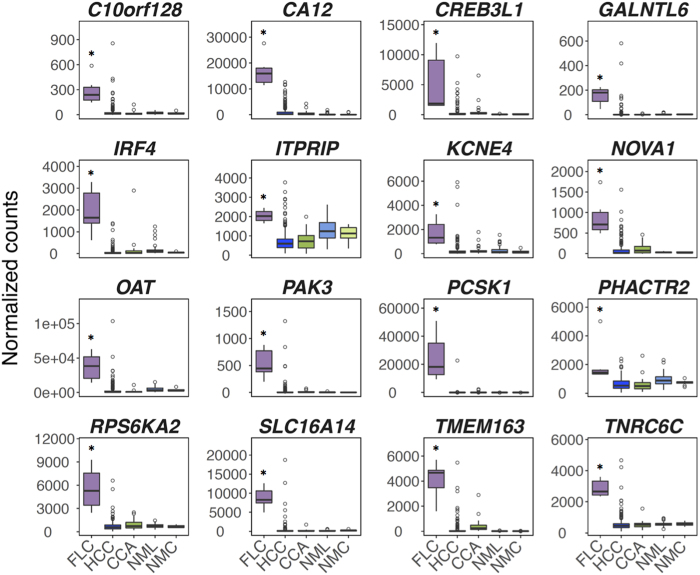
Sixteen genes most uniquely distinguish FLC from other liver cancers. Boxplots depicting RNA levels of 16 genes in the FLC mRNA signature in FLC (n = 6), HCC (n = 263), CCA (n = 36), non-malignant liver (NML, n = 50), and non-malignant cholangiocyte/bile duct (NMC, n = 9) from TCGA. Y-axis shows counts normalized by DESeq. Shaded regions of boxplots show the 25^th^–75^th^ quantiles of the data with the median denoted by a bold line. Whiskers of boxplots represent data <25^th^ and >75^th^ quantiles. Circles represent data points that are outliers, defined as points <25^th^ quantile minus 1.5*IQR (interquartile range, 75^th^–25^th^ quantile) or >75^th^ quantile plus 1.5*IQR. *FDR < 0.05 (DESeq, negative binomial test) of FLC compared to both HCC and CCA.

We next sought to determine which among the 163 genes are most unique to FLC compared to HCC and CCA. Therefore, we identified the genes whose expression levels in all FLC samples are greater or less than 95% of all HCC and CCA samples. This analysis resulted in a set of 16 genes, which we refer to as the FLC mRNA signature (Fig. 3). All 16 of these genes are up-regulated in FLC and also distinguish FLC tumors from non-malignant liver and non-malignant cholangiocytes (Fig. 3). We validated this FLC gene signature in two independent cohorts of FLC cases. Using RNA-seq data for 20 FLC tumors from Honeyman *et al*.^17^ we confirmed that 14 out of the 16 genes (except *CREB3L1* and *ITPRIP*) are indeed significantly elevated in FLC relative to HCC and CCA (Supplementary Fig. S7). Analysis of microarray data for 34 FLC tumors from Cornella *et al*.^11^ demonstrated that most of these genes were significantly elevated in FLC compared to non-malignant liver tissue (Supplementary Fig. S8). Further analysis of the Cornella *et al*. dataset, in which the authors had provided evidence for three different FLC subtypes^11^, showed that several genes (e.g., *CA12, NOVA1, PCSK1, TMEM163*, and *TNRC6C*) are significantly elevated in FLC irrespective of the subtype (Supplementary Fig. S9).

CK7 and CD68 are currently used as protein markers to diagnose FLC^3^. However, CK7 protein is expressed in approximately 1/3 of conventional HCCs^33^ and CD68 is routinely expressed in macrophages located within the sinusoids of conventional HCCs^34^. In fact, our analysis shows that at least at the RNA level, many HCCs and CCAs express both *CD68* and *CK7* at levels observed in FLCs (Supplementary Fig. S10). Furthermore, many other current and proposed markers of FLC^3^,^13^,^14^,^15^,^26^,^27^,^30^,^35^ do not seem to effectively distinguish between FLC and other liver cancers (Supplementary Fig. S11). Two genes in the 16-gene signature, protein convertase subtilisin/kexin type 1 (*PCSK1*) and p21-activated kinase 3 (*PAK3*), were previously described as elevated in FLC by multiple studies^13^,^14^,^15^. Both *PCSK1*^36^ and *PAK3*^37^ have been reported as markers of neuroendocrine tumors and FLC tumors have been shown to display neuroendocrine characteristics^13^,^14^. However, notably, 14 out of the 16 genes in the signature have not been linked etiologically to FLC or specifically highlighted previously as biomarkers of FLC relative to other tumor types.

### Analysis of the FLC mRNA signature across non-liver tumor types

**Figure 4:**
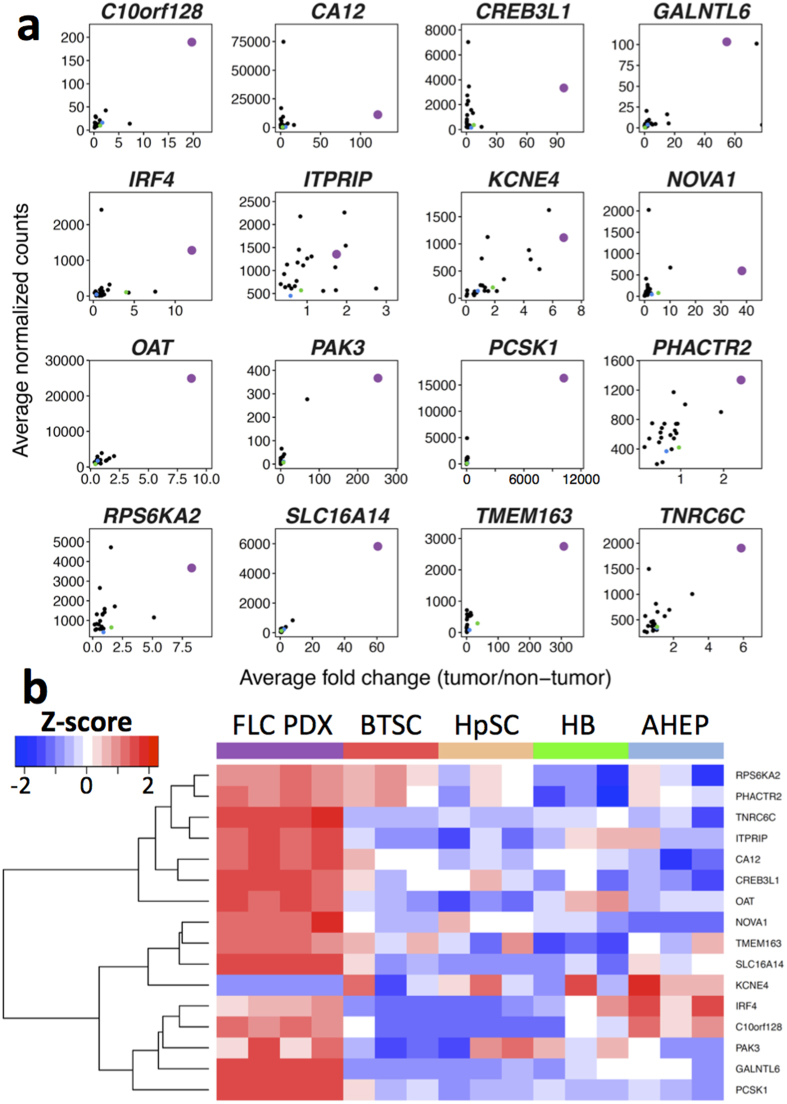
The FLC mRNA signature distinguishes FLC from non-liver cancers and normal liver maturational lineage stages. (**a**) Scatterplot where each point represents a different tumor type within TCGA (n = 22). FLC is shown in purple, HCC is shown in blue, and CCA is shown in green. All other tumor types are shown in black. The x-axis displays the average fold change for each tumor type relative to the appropriate non-tumor tissue and the y-axis displays the average RNA levels of each gene in each tumor type. (**b**) Heatmap showing the RNA expression of 16 genes in the FLC mRNA signature in a FLC patient-derived xenograft (PDX) model and four normal maturational lineage stages of the human liver: biliary tree stem cells (BTSC), hepatic stem cells (HpSC), hepatoblasts (HB), and adult hepatocytes (AHEP). Shown are n = 4 distinct passages of the FLC PDX model and n = 3 biological replicates for each liver lineage stage.

We next sought to determine if these 16 genes could also distinguish FLC from other non-liver cancers. We found by analysis of RNA-seq data in TCGA, for 7211 tumor samples across 20 non-liver cancer types with available non-tumor tissue data (Supplementary Table S3), that 14 of the 16 genes are up-regulated to a greater extent in FLC relative to the corresponding adjacent non-malignant tissue than in any other tumor type (Fig. 4a). Also, among these 14 genes, eight are more abundant in FLC than in any other tumor type (Fig. 4a), indicating that these eight genes not only distinguish FLC from other liver tumors, but also likely uniquely mark FLC. These eight genes include ornithine aminotransferase (*OAT*) and solute carrier family 16 member 14 (*SLC16A14*), which are on average 6.4 and 7.0 fold more highly expressed in FLC, respectively, than the tumor type with the next highest expression. Six of the 14 genes are not uniquely elevated in FLC, such as neuro-oncological ventral antigen 1 (*NOVA1*) and carbonic anhydrase XII (*CA12*), but several of these have been described as candidate oncogenes in the context of other cancers. For example, *NOVA1* overexpression leads to invasion and proliferation in HCC cell lines and is correlated with poor prognosis in HCC patients^38^. *CA12* regulates extracellular pH and is overexpressed in a subset of renal cell carcinomas and breast tumors^39^,^40^. Additionally, inhibition of CA12 and other carbonic anhydrase family members has been proposed as a cancer therapeutic^41^,^42^,^43^.

We also evaluated by RNA-seq the expression of the 16 genes in our previously described patient-derived xenograft (PDX) model of FLC, which is currently the only FLC disease model available, as well as in normal cells of four different maturational lineage stages of the liver: biliary tree stem cells (BTSC), hepatic stem cells (HpSC), hepatoblasts (HB), and adult hepatocytes (AHEP). We found that 15 of the 16 are dramatically up-regulated in the FLC PDX model compared to all of the other liver lineage stages, including BTSCs (Fig. 4b). Our previous work has suggested that BTSCs may be the cell type of origin for FLC tumors^16^. The finding that most genes in our FLC mRNA signature are elevated in the FLC PDX model compared to BTSCs suggests that they may also play a functional role in FLC pathogenesis.

### Identification of differentially expressed long non-coding RNAs in FLC compared to HCC and CCA

**Figure 5:**
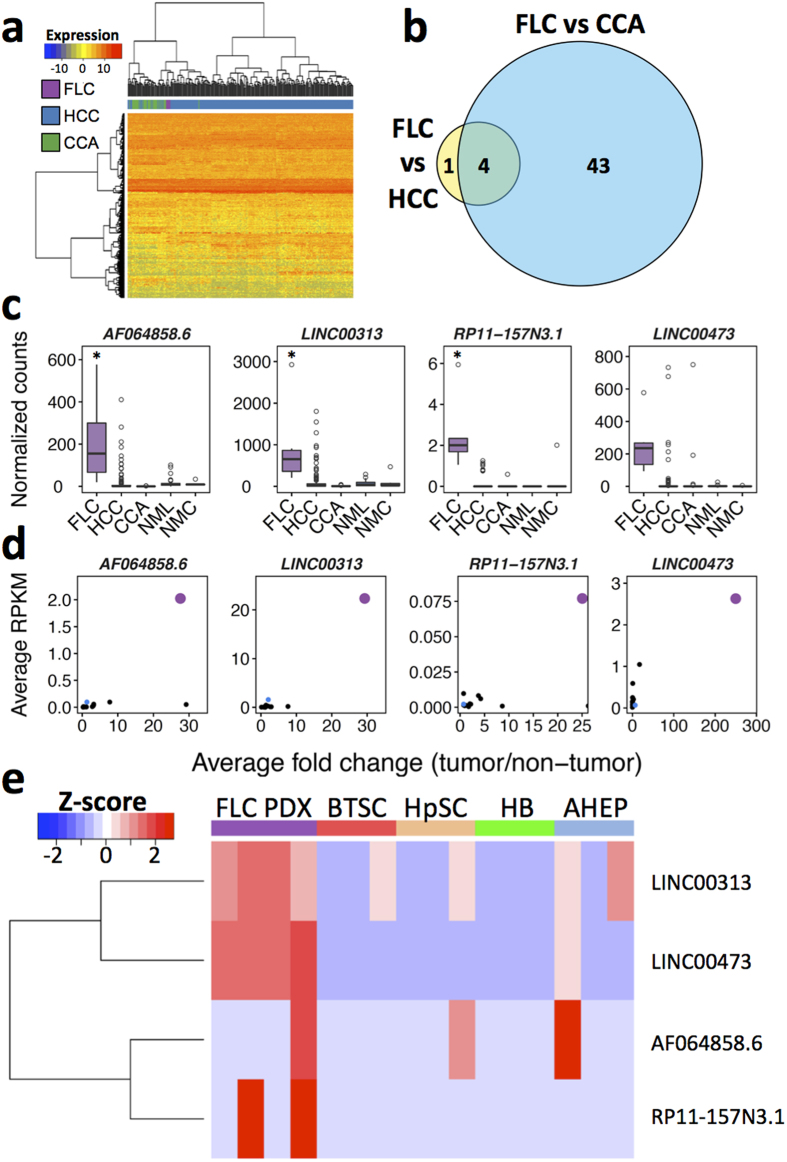
FLCs share a unique lincRNA expression profile. (**a**) Hierarchical clustering of FLC (n = 6), HCC (n = 263), and CCA (n = 36) samples in TCGA. Clustering was performed using the 500 most variable lincRNAs across all tumors following Variance Stabilizing Transformation with Euclidian distance and Ward’s minimum variance method. (**b**) Venn diagram depicting differentially expressed lincRNAs between FLC and both HCC and CCA. Genes were defined as differentially expressed using the following criteria: fold change >2 and FDR < 0.05. (**c**) Boxplots depicting expression of the three members of the FLC lincRNA signature and *LINC00473* in FLC, HCC, CCA, non-malignant liver (NML), and non-malignant cholangiocytes/bile duct (NMC) from TCGA. (**d**) Scatterplot of three members of the FLC lincRNA signature and *LINC00473* where each point represents a different tumor type within TCGA (n = 14), with FLC marked in purple, HCC in blue, and all other tumor types in black. The x-axis displays the average fold change for each tumor type relative to the appropriate non-tumor tissue and the y-axis displays the average expression level of each lincRNA in each tumor type. (**e**) Heatmap showing the expression of three members of the FLC lincRNA signature and *LINC00473* in a FLC patient-derived xenograft (PDX) model and four normal maturational lineage stages of the human liver: biliary tree stem cells (BTSC), hepatic stem cells (HpSC), hepatoblasts (HB), and adult hepatocytes (AHEP). *FDR < 0.05 (DESeq, negative binomial test) of FLC compared to both HCC and CCA.

LincRNAs play an important role in the biology of a wide array of different tumor types^21^, but have never previously been characterized in FLC. To determine if the lincRNA expression profile of FLCs differs from that of other liver cancers, we performed hierarchical clustering of FLC (n = 6), HCC (n = 263), and CCA (n = 36) samples with TCGA RNA-seq data using the 500 most variable lincRNAs across all samples. Our analysis revealed that the six FLCs clustered together in a clade separate from all HCCs and CCAs (Fig. 5a), indicating that FLCs have a unique lincRNA expression profile distinct from other liver cancers.

To identify specific lincRNAs that are differentially expressed in FLC compared to both HCC and CCA, we performed differential lincRNA expression analyses using DESeq. LincRNAs were defined as differentially expressed if they exhibited >2 fold-difference in average expression level and FDR < 0.05 between FLC and both HCC and CCA. We found five differentially expressed lincRNAs between FLC and HCC and 47 between FLC and CCA. Of these, 4 were differentially expressed between FLC and both HCC and CCA with concordant directionality (Fig. 5b, Supplementary Table S4).

### Determination of an FLC lincRNA signature

We next sought to determine which among the 4 lincRNAs are most unique to FLC compared to HCC and CCA. Therefore, we identified the lincRNAs whose expression levels in all FLC samples are greater or less than 95% of all HCC and CCA samples. This analysis identified one lincRNA, *RP11-157N3.1*. When we relaxed the threshold from 95% to 90%, we identified a total of three lincRNAs (*AF064858.6, LINC00313, RP11-157N3.1*), which we refer to as the lincRNA signature (Fig. 5c). The robust up-regulation of these three lincRNAs in FLC relative to HCC was validated using an independent database of quantified lincRNAs called The Atlas of Non-coding RNAs in Cancer (TANRIC) (Supplementary Fig. S12). Interestingly, *LINC00313* was previously annotated as the potential protein-coding gene *C21orf84* and was identified as differentially expressed between FLC and both HCC and CCA in the mRNA analysis above (Fig. 2b, Supplementary Table S2). Additionally, we noticed that while another lincRNA, *LINC00473*, missed the threshold for significance, it was nevertheless notably overexpressed in FLC (Fig. 5c). *LINC00473* (previously annotated as *C6orf176*) has been linked to cAMP-mediated gene regulation^44^,^45^,^46^. Since FLCs express a recurrent fusion protein containing the catalytic subunit of PKA and PKA is a cAMP-dependent protein kinase, *LINC00473* may serve as a marker of aberrant cAMP levels or as a downstream effector of cAMP-mediated mechanisms.

### Analysis of the FLC lincRNA signature across non-liver tumor types

We next sought to determine if these four lincRNAs could also distinguish FLC from other non-liver cancers. We found by analysis of data in the TANRIC database that all four of the lincRNAs are up-regulated to a greater extent in FLC relative to the corresponding adjacent non-malignant tissue than in any other tumor type (Fig. 5d). Furthermore, all four are more abundant in FLC than in any other tumor type (Fig. 5d), suggesting that these lincRNAs not only distinguish FLC from other liver tumors, but are also likely unique to FLC. Finally, we also evaluated by RNA-seq the expression of the four lincRNAs in the FLC PDX model as well as in each of the four different maturational lineage stages of the liver mentioned above. Only the levels of *LINC00313* and *LINC00473* were significantly elevated in the FLC PDX model (Fig. 5e).

### Validation of CA12 overexpression in FLC

**Figure 6:**
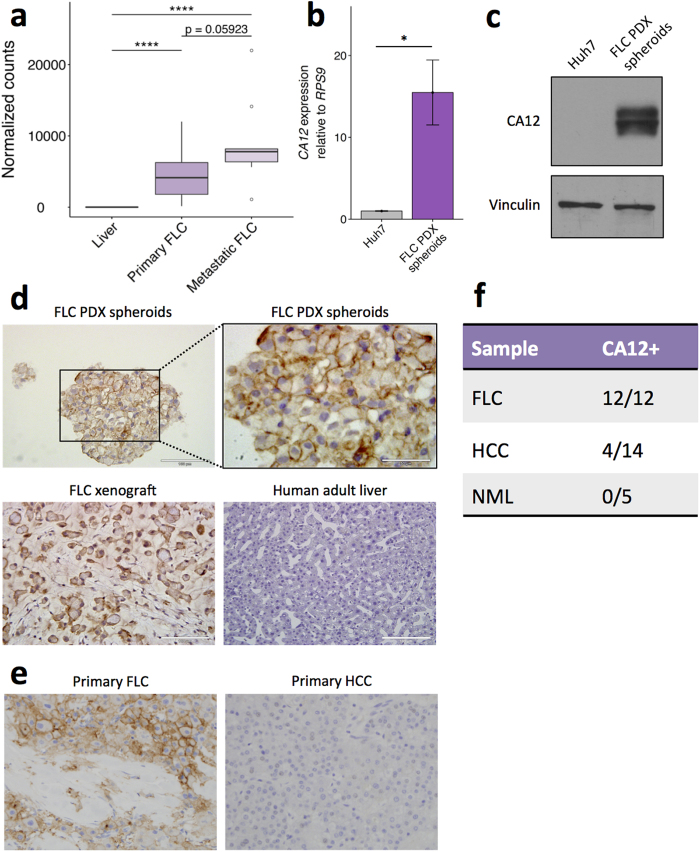
Validation of aberrant CA12 upregulation in FLC. (**a**) *CA12* RNA levels quantified by RNA-seq in non-tumor adjacent liver, primary FLC tumors, and metastatic FLC tumors in an independent cohort of FLC tumors^17^. (**b**) *CA12* expression quantified by qRT-PCR in cultured spheroids from a FLC PDX model and Huh7, a human HCC cell line. *CA12* expression was normalized to *RPS9*. Statistical significance determined by Student’s two-tailed t-test. Data shown are mean ± SD and display one representative experiment of biological replicates (n = 3 for Huh7, n = 6 for FLC PDX spheroids) of 4 total experiments. (**c**) CA12 expression measured by western blot in cultured spheroids from a FLC PDX model and Huh7. Uncropped blots are presented in Supplementary Fig. S14. (**d**) CA12 expression measured by immunohistochemistry in cultured spheroids from a FLC PDX model, xenograft tissue from a FLC PDX model, and normal human adult liver tissue. Magnification, 40x (top right), 20x (rest). (**e**) CA12 expression measured by immunohistochemistry in primary FLC and HCC. Magnification, 20x. (**f**) Table showing summary of samples with CA12 positive staining by IHC. NML, non-malignant liver. *p < 0.05, ****p < 0.0001 (Mann-Whitney U test).

Lastly, we aimed to provide comprehensive validation of one of the genes in the FLC signature. We selected *CA12* for four reasons. First, CA12 upregulation has already been associated with other highly aggressive cancers, most notably specific subtypes of renal cell carcinoma and breast cancer^39^,^40^. Second, CA12 regulates extracellular pH, which plays a role in important cancer processes including invasion and metastasis. Third, consistent with the known functions of CA12, we observed substantially greater mRNA levels of *CA12* in metastatic FLC tumors compared to primary FLC tumors in an independent dataset^17^ (Fig. 6a). Fourth, PKA has been shown to phosphorylate CA9, a carbonic anhydrase in the same family as CA12 with similar function and cellular localization^47^. Phosphorylation of CA9 modulates its activity, implicating PKA in tumor invasion in part by regulation of carbonic anhydrases and extracellular pH.

We first validated overexpression of CA12 in our FLC PDX model at the RNA and protein level compared to Huh7 cells, a human HCC cell line. RT-qPCR and western blot analyses showed significant upregulation of CA12 RNA and protein expression, respectively, in FLC cells (Fig. 5b,c). Importantly, RT-qPCR detected the expression of DNAJB1-PRKACA transcripts in our FLC PDX model, but not in Huh7 cells (Supplementary Fig. S13). Immunohistochemistry (IHC) of cells from our FLC PDX model grown as spheroids and FLC xenografts showed dramatic upregulation of CA12 compared to normal liver (Fig. 5d). Additional IHC staining of primary samples revealed positive signal for CA12 in 12/12 FLC cases, but only 4/14 HCC tumors, and 0/5 normal liver samples (Fig. 5e,f).

## Discussion

FLC is a rare liver cancer that primarily affects adolescents and young adults^1^. Previous genomic studies have primarily compared FLC tumors to adjacent non-tumor tissue^10^,^11^,^13^,^15^,^17^. However, global transcriptomic comparisons of FLC to multiple other liver cancers, or other non-liver tumor types, have not been examined. Here, through RNA-seq analyses of protein coding genes and lincRNAs in FLC, HCC, and CCA, as well as >25 other tumor types, we identify a unique mRNA and lincRNA signature and candidate oncogenes in FLC.

Recently, multiple studies have confirmed the presence of a fusion transcript, *DNAJB1-PRKACA*, in FLC tumor samples^11^,^13^,^16^,^17^. This fusion has been proposed to be specific to FLC^20^. Examining nearly 10,000 samples, we have performed the most extensive analysis evaluating this hypothesis and find that *DNAJB1-PRKACA* is indeed specific to FLC tumors. This finding motivates further implementation of FLC diagnostics testing for the presence of this unique fusion. Although *DNAJB1-PRKACA* is present in the majority of FLC tumors, one group recently reported FLC tumors lacking the chimera^11^. It remains unclear if these tumors really lack the chimera, if the chimera is expressed at lower levels, or if these tumors are misclassified. If there is indeed a minority of FLC tumors without the fusion, further work is necessary to determine whether these cancers resemble the majority of FLC tumors at the transcriptomic level.

In our analyses, we detected robust evidence of *DNAJB1-PRKACA* expression in six tumor samples all classified as Liver Hepatocellular Carcinoma. Interestingly, three samples were subannotated as FLC, while three were subannotated as HCC. For these six samples, we confirmed the presence of the chromosome 19 deletion that results in the fusion transcript through WES or SNP array. The ages of all six patients (17–34) were also consistent with those previously reported for FLC patients (Supplementary Fig. S1). Evaluation of the single histology slide available for each tumor demonstrated that four tumors (three subannotated as FLC and one subannotated as HCC) displayed classical FLC histology, while two tumors (both subannotated as HCC) displayed histological features of both FLC and HCC. Unfortunately, without access to these samples or additional histological images, we were unable to perform diagnostic stains or evaluation to further confirm the FLC diagnosis. Importantly, both samples with and without classical FLC histology showed similar evidence for the presence of the *DNAJB1-PRKACA* fusion transcript.

FLC tumors with regions of histology resembling HCC have previously been identified. Notably, in one case fluorescent *in situ* hybridization identified genomic PRKACA rearrangements throughout the tumor including the regions with HCC histology^20^. Previous work has also demonstrated that FLCs with classical histology have distinct gene expression signatures than FLCs with mixed histology^14^. While our results demonstrate that all six FLCs shared similar gene expression profiles, we did notice that the two FLC tumors with mixed histology consistently clustered together (data not shown), confirming the previous findings. While FLCs with mixed histological features have been previously described^14^,^20^, more work is necessary to determine whether these are truly distinct etiologically from FLCs with classical histology.

We identified 163 genes using DESeq that were differentially expressed in FLC compared to both HCC and CCA (Fig. 2b). These genes also distinguished FLC from 20 additional non-liver tumors (Supplementary Fig. S6). However, it is worth noting that we did identify a small subset of papillary renal cell carcinoma (KIRP) samples that displayed similar gene expression signatures to FLC and were enriched for type 2 KIRP tumors^48^. Interestingly, type 2 KIRP tumors are highly aggressive like FLC and also share some very similar histological features with FLC including large cells with eosinophilic cytoplasm and prominent nucleoli^48^. Gene ontology analysis of these genes revealed enrichment of multiple types of kinase activity and growth factor binding (Fig. 2c). Additionally, we performed kinase enrichment analysis^49^, which identifies enrichment for substrates, curated from multiple kinase-substrate resources, in a given gene set. Kinase enrichment analysis of these 163 genes demonstrated enrichment of PRKACA substrates (Fig. 2d) suggesting that PKA activity, likely though the DNAJB1-PRKACA fusion, distinguishes FLC from both HCC and CCA. Within this set of 163 genes, we identified 16 genes that most uniquely distinguished FLC from other liver cancers that we refer to as the FLC mRNA signature (Fig. 3). Notably, only 2 of the genes in the mRNA signature, *PCSK1* and *PAK3*, have been previously described as markers of FLC^13^,^14^,^15^.

Apart from *PCSK1* and *PAK3*, none of the remaining 14 genes have been explicitly linked to FLC. However, some of these have been shown to play important biological functions in other cancers. For example, *NOVA1* encodes for a RNA-binding protein that is associated with poor overall survival and increased recurrence in HCC patients. Overexpression of NOVA1 also increases HCC proliferation, invasion, and migration^38^. *CA12* codes for a carbonic anhydrase responsible for regulating extracellular pH and is elevated in certain subsets of renal cell carcinoma and breast cancers^39^,^40^. Inhibition of CA12 enzymatic activity has also been proposed as a therapeutic strategy for breast cancer^41^,^42^,^43^. Additionally, some of the remaining genes in the FLC mRNA signature including, *TMEM163, TNRC6C*, and *C10orf128* have yet to be extensively characterized in cancer. Future studies will be necessary to determine if they play a functional role in FLC biology.

We evaluated the expression of the 16 genes in the mRNA signature in 20 non-liver tumor types within TCGA and found that 14 of the 16 genes are more up-regulated in FLC relative to the corresponding adjacent non-malignant tissue than in any other tumor type (Fig. 4a). Among these 14 genes, eight are more abundant in FLC than in any other tumor type, demonstrating that these eight genes not only distinguish FLC from other liver tumors, but also likely uniquely mark FLC. Among these eight genes are *OAT* and *SLC16A14*. OAT, ornithine aminotransferase, is a mitochondrial protein that catalyzes the reversible formation of proline from ornithine. *OAT* is positively regulated by β-catenin and cAMP and inhibition of OAT in HCC suppresses proliferation^50^,^51^,^52^. SLC16A14 is a poorly characterized monocarboxylate transporter suggested to play a role in resistance to chemotherapy in ovarian cancer^53^.

Our previous work has demonstrated that FLCs are most similar to biliary tree stem cells (BTSCs) compared to three other maturational lineage stages of the liver based on gene expression profile^16^. These results suggest that BTSCs may be the cell type of origin for FLC tumors. Interestingly, FLC tumors do not cluster with cholangiocarcinomas (Fig. 2a), which are commonly thought to derive from normal biliary cells. Examination of the 16 genes in the mRNA signature in a unique FLC patient-derived xenograft (PDX) model and these four different lineage stages of the liver, including BTSCs, demonstrated upregulation of 15 genes in FLC compared to all other lineage stages (Fig. 4b).

Non-coding RNAs play a major role in many biological processes, including cancer initiation and progression^21^,^24^. However, non-coding RNAs have yet to be explored in the context of FLC. We decided to focus on long intergenic non-coding RNAs (lincRNAs), which have been shown to be important in proliferation and metastasis. Hierarchical clustering of FLC, HCC, and CCA samples based on the 500 most variable lincRNAs demonstrated that FLCs share a distinct lincRNA expression profile compared to other liver cancers (Fig. 5a). We identified four lincRNAs that were differentially expressed between FLC and both HCC and CCA (Fig. 5b) and found that three of these were more highly expressed in all FLC samples than 90% of the HCC and CCA samples (Fig. 5c). Additionally, we identified one lincRNA, *LINC00473*, which missed the significance threshold, but was still overexpressed in FLC. We refer to these four as the FLC lincRNA signature. Interestingly, *LINC00473* expression is positively regulated by cAMP through PKA and has been suggested as a biomarker of deregulated cAMP signaling^44^,^45^. Very recently it was shown in non-small cell lung cancer (NSCLC) that *LINC00473* is regulated by PKA/CREB, correlates with poor prognosis, and is required for tumor growth and survival. Our analysis confirms that *LINC00473* is highly expressed in NSCLC; however, remarkably, the levels are 3-fold higher in FLC. These data indicate that the possibility of a mechanistic role for *LINC00473* in FLC pathogenesis merits further investigation. The four lincRNAs in the signature also are more abundant in FLC than in multiple non-liver tumors as well as more overexpressed compared to adjacent non-tumor tissue in FLC than other tumor types (Fig. 5d). Evaluation of the lincRNA signature demonstrated overexpression of two lincRNAs, *LINC00313* and *LINC00473*, in FLC compared to four lineage stages of the liver, including BTSCs, suggesting these two lincRNAs may play a role in FLC pathogenesis (Fig. 5e).

Finally, we validated overexpression of one gene in the FLC mRNA signature, CA12. At both the RNA and protein level, CA12 was upregulated in FLC compared to HCC and normal liver (Fig. 5). Interestingly, we also observed high CA12 expression in metastatic FLC tumors, consistent with previous reports suggesting that CA12 and extracellular pH play an important role in metastasis^40^.

We have identified a signature of protein-coding genes and lincRNAs that distinguish FLC from ~22 other liver and non-liver cancers. Additional analysis has revealed that these genes are upregulated in FLCs compared to BTSCs, the potential FLC cell type of origin, suggesting that many of these genes may play a role in FLC pathogenesis. Functional evaluation of these genes will be necessary to dissect their role(s) in FLC. These genes also provide additional confirmatory evidence for the diagnosis of FLC. Whether these genes are upregulated in FLC independent of the DNAJB1-PRKACA fusion or as a result of it remains uncertain. In either situation, these genes may act to promote development and/or progression of FLC. If there do exist true FLCs lacking the *DNAJB1-PRKACA* fusion, it remains unclear if these genes are also upregulated in these tumors.

Overall, our results point to dysregulation of cAMP/PKA signaling as a major force shaping the gene expression landscape in FLC and also reveal additional genes that may play an important role in FLC etiology. Functional validation of the DNAJB1-PRKACA fusion and genes in the mRNA and lincRNA signature in the context of FLC may reveal novel therapeutic targets of this rare and deadly cancer.

## Materials and Methods

### RNA expression analysis

RNA-seq data for TCGA fusion (9840 samples—9148 tumor and 691 non-tumor samples—across 29 annotated tumor types, Supplementary Table S1) and differential gene expression analysis (263 HCC, 6 FLC (both HCC and FLC are annotated as LIHC within TCGA), 50 non-tumor liver, 36 CCA, 9 non-tumor bile duct/cholangiocytes) were downloaded from the Cancer Genomics Hub (CGHub). RNA-seq data for one FLC validation cohort (20 FLC, 9 non-malignant liver)^17^ were downloaded from the database of Genotypes and Phenotypes (dbGaP, study accession phs000709.v1.p1). Microarray data for a second FLC validation cohort (34 FLC, 5 non-malignant liver)^11^ were downloaded from the Gene Expression Omnibus (GEO, GSE57725). RNA-seq data for the FLC PDX (patient-derived xenograft) model and liver lineages were previously described^16^ and are available on GEO (GSE73114). Normalized (quartile normalization) data for gene expression analysis across 20 non-liver tumors, LIHC, and CCA (8302 samples—7621 tumor and 681 non-tumor samples, Supplementary Table S3) were obtained from the TCGA Data Portal. Normalized (RPKM) data for lincRNA expression analysis across 13 non-liver tumors and LIHC (5367 samples—4803 tumor and 564 non-tumor samples, Supplementary Table S5) were obtained from The Atlas of non-coding RNA in cancer (TANRIC)^54^. Quality of FASTQ files was assessed using FastQC and reads were aligned to the human hg19 genome with MapSplice2^55^. Transcripts were quantified using RSEM^56^ with 2011 UCSC Known Gene definitions for protein-coding genes and GENCODE release 19 for lincRNAs. Normalization and differential expression analysis for RNA-seq were performed using DESeq^57^. Genes were classified as differentially expressed if they met the following criteria: fold change ≥2, false discovery rate (FDR) < 0.05, and >50 normalized counts in at least one tissue type. LincRNAs were classified as differentially expressed if they met the following criteria: fold change ≥2 and FDR < 0.05. Hierarchical clustering was performed in R using Euclidean distance and Ward’s minimum variance method following Variance Stabilizing Transformation provided by DESeq. Gene ontology and kinase enrichment analysis^49^ was performed using Enrichr^58^. Differential expression for microarray data was performed using the Mann-Whitney U test.

### Copy number variation analysis

For whole exome sequencing analysis, 10 kb bin counts data were generated using BEDTools. The read ratios were calculated by using the count in tumor divided by the count in matched normal for each bin. A circular binary segmentation^59^ procedure with default parameters was applied to segment the genome. For SNP array analysis, publically available level 3 segmented copy number data for all TCGA LIHC Affymetrix 6.0 SNP arrays were downloaded through the Broad Institute’s TCGA GDAC Firehose data portal (http://gdac.broadinstitute.org/). We filtered for segments that included a copy number alteration on chromosome 19. Samples with a deletion at chr19:14239803-14624494 were identified by having a segment annotation (i.e. segment start and segment stop genomic position) that completely encompasses chr19:14239803-14624494 and that also have a segment mean less than or equal to −0.1.

### Histology and patient ages

Histology images were accessed from the TCGA Data Portal and from the Cancer Digital Slide Archive^60^. Liver histology was reviewed independently by two gastrointestinal pathologists (KGG, MST). Ages of patients were obtained from TCGA using the R package TCGA2STAT^61^.

### Cells

Cells from the patient-derived xenograft (PDX) model of FLC were grown in *NOD.Cg-Prkdc*^*scid*^ *Il2rg*^*tm1Wjl*^/*SzJ* or NOD scid gamma (NSG) immunocompromised mice, isolated, and cultured as previously described^16^. Procedures were performed according to protocols approved by the UNC School of Medicine at Chapel Hill IACUC. The mice were housed in UNC’s DLAM sterile facility in micro-isolated autoclaved cages with free access to autoclaved water and radiation sterilized food. Huh7 cells were obtained and cultured as previously described^62^.

### Primary samples

12 FFPE fibrolamellar carcinomas, 14 hepatocellular carcinomas, and 3 non-malignant livers were collected from the Mayo Clinic (Rochester, MN) institutional clinical archives. 2 additional non-malignant livers were obtained from human donors. The FLC samples were previously shown to harbor *DNAJB1-PRKACA* transcripts by qRT-PCR and were positive for a PRKACA rearrangement by FISH^20^. We have characterized these cases as morphologically typical fibrolamellar carcinomas and they were previously part of another study, which showed that FGFR1 was not amplified in fibrolamellar carcinoma^63^.

### Quantitative real-time PCR

qRT-PCR was carried out as described previously^62^,^64^. Briefly, total RNA was extracted using the Total RNA Purification Kit (Norgen Biotek, Thorold, Ontario, Canada). Reverse Transcription was performed using the High Capacity RNA-to-cDNA Kit (Thermo Fischer Scientific, Waltham, MA). Gene expression was quantified with using TaqMan Gene Expression Assays (Thermo Fischer Scientific) on a CFX96 Touch Real-Time System (Bio-Rad, Hercules, CA). Assays were performed in triplicate. *CA12* and *DNAJB1-PRKACA* mRNA expression levels were normalized to the housekeeping gene, *RPS9. CA12* fold increase was calculated based on the 2^−ΔΔCT^ method in comparison to Huh7 cells, a human HCC cell line. The Taqman assays used were *CA12* (Assay ID Hs01080902_m1) and *DNAJB1-PRKACA* (custom assay, F: CGCAAGCGCGAGATCTTC, R: GAAAATCTTCTTTGGCTTTGGCTAAGA, Probe: CTTTCACTTCCTCCCCGTAGCG).

### Western blotting

Huh7 cells and FLC PDX spheroids were lysed in lysis buffer (RIPA buffer (Sigma, St. Louis, MO), 25x Complete Protease Inhibitor Cocktail (Sigma), 100x Pierce Phosphatase Inhibitor (Thermo Fisher Scientific), 100 mM phenylmethanesulfonyl fluoride, β-mercaptoethanol, 1 M dithiothreitol) at 4 °C. All lysates were flash frozen, thawed, and centrifuged for 10 minutes at 14,000 × g at 4 °C. Protein concentration was quantified using the Pierce BCA Protein Assay Kit (Thermo Fisher Scientific). Samples were diluted 1:1 in Laemmli Sample Buffer (Bio-Rad) containing 5% β-mercaptoethanol, heated at 95 °C for 3 minutes, loaded into 12% Mini-PROTEAN TGX Precast Gel (Bio-Rad), and run in 1X Tris/Glycine/SDS buffer (Bio-Rad) for 75 minutes at 150 V. Transfer was performed with the Trans-Blot Turbo Transfer System (Bio-Rad). Membranes were blocked with either 4% milk or bovine serum albumin (BSA), probed with primary mouse anti-CA12 (1:500, ab140385, Abcam, Cambridge, MA) or rabbit anti-vinculin (1:1000, #4650, Cell Signaling Technology, Danvers, MA), and then incubated with horseradish peroxidase conjugated secondary antibodies. Membranes were incubated in ECL Prime Western Blotting Detection System (GE Healthcare, Little Chalfont, UK) for 5 minutes before visualization.

### Immunohistochemistry

FLC xenograft tissue, FLC PDX spheroids, and non-malignant liver (n = 2) were fixed in 4% paraformaldehyde overnight and stored in 70% ethanol. Samples were paraffin embedded, cut into 5 μm sections, and deparaffinized. Antigen retrieval was performed by steaming tissue sections in 1x sodium citrate buffer, Citrate Plus (ScyTec, West Logan, UT) for 20 minutes. Endogenous peroxidase activity was blocked using 3% hydrogen peroxide diluted in TBS for 15 minutes at room temperature. Endogenous biotin activity was blocked using 2.5% Normal Horse Serum Blocking Solution (Vector Laboratories, Burlingame, CA) for 1 hour at room temperature. Sections were incubated in primary mouse anti-CA12 (1:75, ab140385, Abcam), diluted in TBS, overnight at 4 °C. Sections were then incubated in secondary antibody, ImmPRESS™ REAGENT Anti-mouse Ig (Vector Laboratories, #MP-7402) at room temperature for 30 minutes. Chromogen staining was performed using ImmPACT DAB Peroxidase Kit (Vector Laboratories, #SK-4105). Sections were lightly counterstained using hematoxylin, dehydrated, mounted in xylene, and visualized. For primary FLC, primary HCC, and non-malignant liver (n = 3) samples, tissue sectioning and IHC staining was performed at the Pathology Research Core (Mayo Clinic, Rochester, MN) using the Leica Bond RX stainer (Leica, Buffalo, IL). Formalin Fixed Paraffin Embedded (FFPE) tissues were sectioned at 5 microns. The tissue slides were dewaxed and retrieved on-line using the following reagents: Bond Dewax (Leica) and Epitope Retrieval 2 (EDTA; Leica). Tissue slides were retrieved for 20 minutes. The primary mouse anti-CA12 (ab140385, Abcam) was used at 1:500 and it was incubated for 15 minutes. The detection system used was Polymer Refine Detection System (Leica). This system includes the hydrogen peroxidase block, secondary antibody polymer, DAB, and Hematoxylin. Once completed, slides were removed from the stainer and rinsed for 5 minutes in tap water. Slides were dehydrated in increasing concentrations of ethyl alcohol and xylene prior to permanent coverslipping in xylene based media.

## Additional Information

**How to cite this article**: Dinh, T. A. *et al*. Comprehensive analysis of The Cancer Genome Atlas reveals a unique gene and non-coding RNA signature of fibrolamellar carcinoma. *Sci. Rep.* **7**, 44653; doi: 10.1038/srep44653 (2017).

**Publisher's note:** Springer Nature remains neutral with regard to jurisdictional claims in published maps and institutional affiliations.

## Supplementary Material

Supplementary Information
