# Supplementary figures and images for: Comprehensive analysis of The Cancer Genome Atlas reveals a unique gene and non-coding RNA signature of fibrolamellar carcinoma
Source: Sci Rep. 2017 Mar 17;7:44653. doi: 10.1038/srep44653 (PMC5356346; doi:10.1038/srep44653)

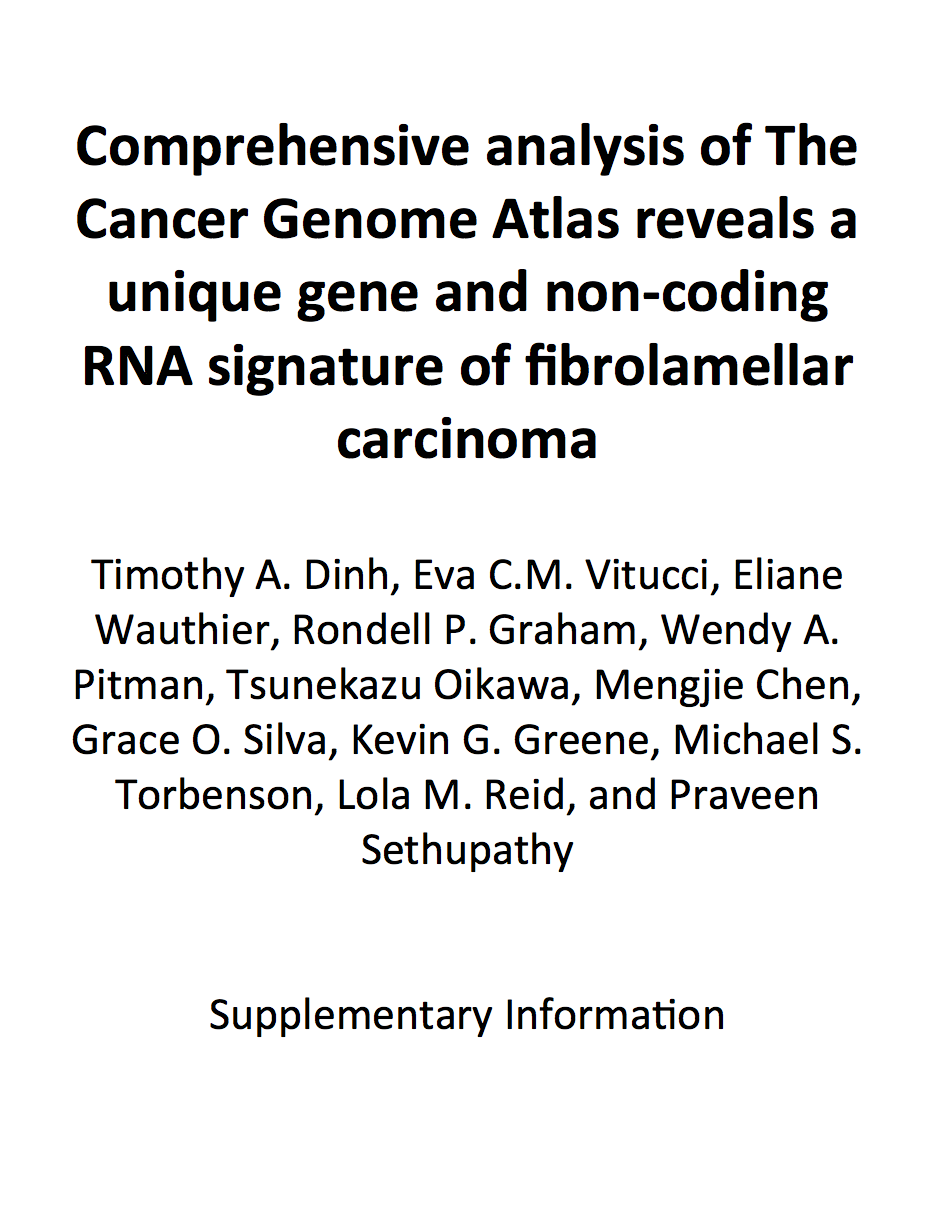

Supplement: Supplementary Information [file srep44653-s1.tiff]
